# Cortical structural changes after subcortical stroke: Patterns and correlates

**DOI:** 10.1002/hbm.26095

**Published:** 2022-10-03

**Authors:** Jingchun Liu, Caihong Wang, Wen Qin, Hao Ding, Yanmin Peng, Jun Guo, Tong Han, Jingliang Cheng, Chunshui Yu

**Affiliations:** ^1^ Department of Radiology and Tianjin Key Laboratory of Functional Imaging Tianjin Medical University General Hospital Tianjin China; ^2^ Department of MRI The First Affiliated Hospital of Zhengzhou University Zhengzhou Henan China; ^3^ School of Medical Imaging Tianjin Medical University Tianjin China; ^4^ Department of Radiology Tianjin Huanhu Hospital Tianjin China; ^5^ CAS Center for Excellence in Brain Science and Intelligence Technology Chinese Academy of Sciences Shanghai China

**Keywords:** brain structure, corticospinal tract, magnetic resonance imaging, stroke, voxel‐based lesion‐symptom mapping

## Abstract

Subcortical ischemic stroke can lead to persistent structural changes in the cerebral cortex. The evolution of cortical structural changes after subcortical stroke is largely unknown, as are their relations with motor recovery, lesion location, and early impairment of specific subsets of fibers in the corticospinal tract (CST). In this observational study, cortical structural changes were compared between 181 chronic patients with subcortical stroke involving the motor pathway and 113 healthy controls. The impacts of acute lesion location and early impairments of specific CSTs on cortical structural changes were investigated in the patients by combining voxel‐based correlation analysis with an association study that compared CST damage and cortical structural changes. Longitudinal patterns of cortical structural change were explored in a group of 81 patients with subcortical stroke using a linear mixed‐effects model. In the cross‐sectional analyses, patients with partial recovery showed more significant reductions in cortical thickness, surface area, or gray matter volume in the sensorimotor cortex, cingulate gyrus, and gyrus rectus than did patients with complete recovery; however, patients with complete recovery demonstrated more significant increases in the cortical structural measures in frontal, temporal, and occipital regions than did patients with partial recovery. Voxel‐based correlation analysis in these patients showed that acute stroke lesions involving the CST fibers originating from the primary motor cortex were associated with cortical thickness reductions in the ipsilesional motor cortex in the chronic stage. Acute stroke lesions in the putamen were correlated with increased surface area in the temporal pole in the chronic stage. The early impairment of the CST fibers originating from the primary sensory area was associated with increased cortical thickness in the occipital cortex. In the longitudinal analyses, patients with partial recovery showed gradually reduced cortical thickness, surface area, and gray matter volume in brain regions with significant structural damage in the chronic stage. Patients with complete recovery demonstrated gradually increasing cortical thickness, surface area, and gray‐matter volume in the frontal, temporal, and occipital regions. The directions of slow structural changes in the frontal, occipital, and cingulate cortices were completely different between patients with partial and complete recovery. Complex cortical structural changes and their dynamic evolution patterns were different, even contrasting, in patients with partial and complete recovery, and were associated with lesion location and with impairment of specific CST fiber subsets.

## Graphical abstract

Complex cortical structural changes and their dynamic evolution patterns were different, even contrasting, in stroke patients with partial and complete recovery, and were associated with lesion location and with impairment of specific corticospinal tract fiber subsets.

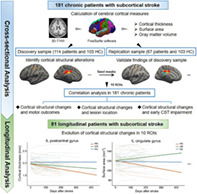

## 1 INTRODUCTION

Subcortical ischemic stroke can lead to structural damage to brain regions adjacent to the stroke lesion and to specific regions of cerebral cortex remote from the lesions (Conrad et al., 2021; Liu et al., 2015), possibly by the mechanism of axonal degeneration (Yu et al., 2009). In response to the structural damage caused by subcortical stoke, some cerebral cortical regions can reorganize themselves to facilitate recovery of the impaired neurological function (Brodtmann et al., 2012; Liu, Peng, et al., 2020). Structural damage and reorganization in the cerebral cortex can be assessed quantitatively in vivo using structural magnetic resonance imaging (MRI) followed by calculation of cortical thickness, surface area, and gray matter volume (GMV) from the imaging data (Gupta et al., 2019). In the past decade, many studies have reported structural alterations in the cerebral cortex in chronic patients with subcortical stroke (Cheng et al., 2020; Diao et al., 2017; Jones et al., 2016; Zhang et al., 2014). However, only a limited number of chronic cortical structural changes reported by one study can be replicated in other studies. The small sample sizes and the lack of independent replication may be one reason for this inconsistency across studies. Thus, the chronic structural changes reliably observed in the cortex after subcortical stroke should be investigated in large samples and replicated in independent data sets.

The location of stroke lesions is another critical factor accounting for some of the observed variation in chronic cortical changes after stroke; stroke lesions in different locations may impair different brain structures and thus result in different cortical changes (Liu, Peng, et al., 2020). This conclusion is supported by a prior study that showed different patterns of brain structural changes between chronic patients with capsular stroke and those with pontine stroke (Jiang et al., 2017). However, details of the correspondence between subcortical stroke locations and the ensuing chronic structural changes in the cortex remain unclear and would be particularly valuable in elucidating the neural mechanism of long‐term behavioral impairment (i.e., motor, cognitive functional) in patients with acute stroke. The technique of voxel‐based lesion‐symptom mapping (VLSM) has been used to establish the correspondence between lesion locations and specific neurological impairments by comparing neurological scores between patients with and without lesions, on a voxel‐by‐voxel basis (Bates et al., 2003; Meyer et al., 2016). This method has had successes in identifying the relationships between stroke lesion locations and motor impairment (Lo et al., 2010), cognitive deficit (Munsch et al., 2016), and aphasia (Harvey & Schnur, 2015; Magnusdottir et al., 2013). Liu et al. found that acute stroke lesions in the bilateral primary motor area (M1) and right supplementary motor area (SMA) fibers were associated with chronic motor deficits (Liu, Wang, et al., 2020). Moreover, in patients with subcortical stroke, the presence of an acute stroke lesion in the right caudate nucleus and nearby white matter was correlated with chronic attention deficit (Liu et al., 2018). Theoretically, the VLSM approach can be adapted to study the correspondence between stroke lesion locations and specific chronic structural changes in the cortex by replacing the neurological scores by cortical structural measurements. However, this approach has not been attempted until now.

The corticospinal tract (CST) is the most important white‐matter tract for motor output in the human brain, and the impairment of the CST has been associated with motor outcomes in patients with subcortical stroke (Lin et al., 2019; Stinear et al., 2007). Although the CST mainly originates from M1, it also contains contributions from the premotor cortex (PMC), SMA, and primary somatosensory area (S1) (Schieber, 2007; Welniarz et al., 2017). Liu, Wang, et al. (2020) have reconstructed a fine‐grained map of the CST fibers showing their different cortical origins, and have associated impairments of different subsets of these CST fibers with different brain structural and functional changes in patients with subcortical stroke. However, the correspondence between early impairment of specific CST fibers and subsequent slow trends in cortical structural parameters has not been systematically investigated in patients with subcortical stroke.

Many studies have associated cortical structural changes with neurological outcomes (Khlif et al., 2021; Ueda et al., 2019) or motor outcomes (Ueda et al., 2019) in patients with subcortical stroke. Subcortical stroke patients with different degrees of recovery appear to have different patterns of cortical structural change in the chronic stage and different trajectories of evolving cortical change poststroke. For example, stroke patients achieving only partial recovery (PR) show more significant reductions in cortical thickness in the primary motor area than do those with complete recovery (CR; Zhang et al., 2014). However, the differences in the evolution patterns of cortical structural changes between stroke patients with different degrees of recovery remain largely unknown, and such information would be helpful for understanding the mechanisms of neurological recovery after subcortical stroke.

In this study, we aimed to (a) identify chronic cortical structural changes in 181 patients with subcortical stroke with a discovery‐replication design and investigate the differences in cortical structural changes between patients with partial and CR; (b) uncover the correspondence of chronic cortical structural changes with acute stroke lesion location, as well as with early impairments of specific CST fiber subsets; and (c) explore the differences in the evolution patterns of cortical structural change between patients with different degrees of recovery in a longitudinal data set of 81 patients with subcortical stroke.

## 2 MATERIALS AND METHODS

### 2.1 Participants

The experimental protocol was approved by the local medical research ethics committee. Written, informed consent was obtained from each participant. This study retrieved cross‐sectional and longitudinal patients with subcortical ischemic stroke from four hospitals. We divided our samples into three independent data sets: the first data set (114 patients with chronic subcortical stroke) was used for discovering chronic cortical structural changes; the second data set (8 independent patients with chronic subcortical stroke) was used for replicating chronic cortical structural changes; and the third data set (81 longitudinal patients with subcortical stroke) was primarily used for observing the longitudinal cortical structural changes. To establish a link between observations of poststroke cortical structural changes, 59 patients with chronic data from data set 3 were also included the replication analysis (8 patients from data set 2 and 59 patients from data set 3).

The inclusion criteria for patients with stroke were as follows: (a) first‐onset acute ischemic stroke; (b) a single lesion in the basal ganglia or neighboring regions; and (c) right‐handedness before stroke onset (Oldfield, 1971). The exclusion criteria were as follows: (a) recurrent stroke defined by clinical history and MRI evaluation; (b) any other brain abnormalities on MR images; (c) a modified Fazekas score for white matter hyperintensities greater than 1 (Fazekas et al., 1987); and (d) history of any other neurological or psychiatric disorders.

In compiling data set 1 and 2, we reviewed the MRI images and clinical data of inpatients within 5 years, and diffusion weighted imaging (DWI) in the acute stage (≤7 days) were used to confirm stroke location. Patients in the chronic stage after stroke onset (>6 months) were included. Patients meeting the inclusion criteria were prospectively recruited to participate in this study. Patients for data set 1 were recruited from August 1, 2014 to March 30, 2015. A total of 123 patients who satisfied the inclusion criteria agreed to participate in this study. Nine patients were excluded for recurrent stroke (*n* = 3), severe white matter hyperintensity (*n* = 3), and history of traumatic brain injury (*n* = 3). Finally, 114 patients for discovery were included in this study. Patients for data set 2 and 3 were recruited from June 1, 2017 to April 30, 2020. A total of 8 patients with subcortical stroke for replication were included in data set 2. In collection of the data set 3, 95 patients with subcortical stroke were recruited followed a longitudinal design (four time points: ≤7 days, 1 month, 3 months and >6 months). Fourteen patients were excluded for loss of follow‐up after inclusion (*n* = 9), recurrent stroke (*n* = 3), and other brain abnormalities (*n* = 2). Finally, 81 longitudinal patients with subcortical stroke (52 patients with data for four time points and 29 patients with data for at least two time points) were included in this study. Healthy controls for data sets 1 and 2 were prospectively recruited from the population sample with the following criteria: (a) sex‐, age‐, and education‐ matched with the patients; (b) free from neurological dysfunction; (c) free from brain structural damage by MRI examination; (d) no history of alcohol or drug dependency; (e) in good physical condition for image acquisition; (f) Fazekas score for white matter hyperintensity ≤1 (Fazekas et al., 1987); and (g) lacunae absent. Finally, 103 cross‐sectional healthy controls (data set 1) and 10 longitudinal healthy controls (data set 2) with data for all the four time points (Supplementary Table 1) were included in this study.

### 2.2 MRI data acquisition

The MRI data of participants were acquired using five 3.0‐Tesla MR scanners from four hospitals, including two Discovery MR750 scanners (General Electric, Milwaukee, WI), two Magnetom Trio Tim MR scanners (Siemens, Erlangen, Germany) and a Signa Excite HDx MR scanner (General Electric, Milwaukee, WI). DWI, T1‐, and T2‐weighted images (T1WI and T2WI), and T2 fluid‐attenuated inversion recovery (T2‐FLAIR) images were acquired to identify stroke lesions, recurrent strokes, white‐matter hyperintensities, and other brain abnormalities. Sagittal 3D T1‐weighted images (3D‐T1WI) were acquired to calculate the cortical structural measures. The DWI parameters were repetition time (TR), echo time (TE), matrix dimensions, field of view (FOV), number of slices, and slice thickness, which were, respectively, 3000 ms, 61 ms, 160 × 160, 240 mm × 240 mm, 20, and 6 mm for the MR 750 scanner; 6000 ms, 95 ms, 128 × 128, 256 mm × 256 mm, 20, and 4 mm for the Trio Tim scanner; and 10,000 ms, 106 ms, 128 × 128, 240 mm × 240 mm, 20, and 6 mm for the Signa Excite HDx scanner. All DWI scans used the same *b*‐value (1000 s/mm^2^). The T2‐FLAIR parameters were TR, TE, matrix, number of slices, and slice thickness, which were, respectively, 8500 ms, 158 ms, 256 × 256, 20, and 5 mm for the MR 750; 10,500 ms, 104 ms, 128 × 128, 20, and 6 mm for the Trio Tim; and 9000 ms, 110 ms, 128 × 128, 20, and 5 mm for the Signa Excite HDx. Sagittal 3D‐T1WI were acquired by brain volume (MR750 and Signa Excite HDx) and magnetization prepared rapid acquisition gradient echo (Trio Tim) sequences with the following imaging parameters: TR, TE, flip angle, matrix, and number of slices, which were, respectively, 8.2 ms, 3.2 ms, 11°, 256 × 256, and 188 for the MR 750 scanner; 2000 ms, 2.3 ms, 9°, 256 × 232, and 192 for the Trio Tim scanner; and 8.1 ms, 3.1 ms, 13°, 256 × 256, and 176 for the Signa Excite HDx. All 3D‐T1WI scans used the same FOV (256 × 256 mm), slice thickness (1 mm, no gap), and voxel size (1 mm × 1 mm × 1 mm). T1WI and T2WI were acquired using clinically accepted sequences (voxel size = 0.5 mm × 0.5 mm × 6 mm).

### 2.3 Neurological assessments

The National Institutes of Health Stroke Scale was used to assess global neurological deficits, and the Fugl‐Meyer Assessment of the whole extremity (WE_FM, i.e., the total score of the upper‐ and lower‐extremity motor assessment) was used to evaluate motor deficits in patients. These assessments were performed in the chronic stage (>6 months) for patients in cross‐sectional data set and at four time points (≤7 days, 1 month, 3 months, and >6 months) for patients in longitudinal data set. For both data sets, patients with subcortical stroke were divided into PR (WE_FM <100) and CR (WE_FM = 100) subgroups according to the WE_FM scores in the chronic stage (>6 months). In 30 patients with PR of longitudinal data set, 13 patients with data for 4 time points and 17 patients with data for at least 2 time points were included. In 51 patients with CR of longitudinal data set, 39 patients with data for 4 time points and 12 patients with data for at least 2 time points were included (Supplementary Table 1).

### 2.4 Calculation of cerebral cortical measures

All structural images (3D‐T1WI) were visually inspected by two radiologists for apparent artifacts due to subject motion and instrument malfunction. Then, the FreeSurfer (FS) V.6.0.0 application (Fischl, 2012) (http://surfer.nmr.mgh.harvard.edu/) was used to preprocess brain structural images and calculate maps of cortical thickness, surface area, and GMV for each participant. The preprocessing procedures were performed using the automated surface‐based pipeline with default parameters, which mainly included segmentation, surface reconstruction, and surface‐based spatial registration. Specifically, structural images were registered to the Talairach atlas, and intensity variation in the white matter was removed by intensity normalization. The skull data were stripped using a deformable template model (Segonne et al., 2004), and white matter was segmented based on intensity and neighbor constraints. The automated analysis pipeline can fail in the presence of the severe structural abnormalities common in stroke (Fischl, 2012). Therefore, to satisfy our predetermined criteria, quality control was performed using Freeview (a visualization tool packaged with FS), involving visual inspection of the segmentation processes by two radiologists with more than 9 years of experience. Then, we manually corrected inaccurate segmentation to improve the FS segmentation results. The gray–white matter surface was obtained by tessellating the gray–white matter boundary and topology correction, then the pial surface was generated by nudging the gray–white matter surface along the T1 intensity gradients to reach the boundary between gray matter and cerebrospinal fluid. Both surfaces were represented by vertices. For each vertex, the distance between the gray–white matter surface and its corresponding pial surface was defined as the cortical thickness (Fischl & Dale, 2000). Then, the cortical thickness, surface area, and GMV at each vertex were obtained for each participant. The resulting maps of cortical measures of each participant were transformed into an average surface space (fsaverage template, provided in the FS package) using a spherical registration method (Fischl et al., 1999), then a 10‐mm full‐width‐at‐half‐maximum Gaussian spatial smoothing kernel was applied to the surface to improve the signal‐to‐noise ratio. These maps were used for vertex‐based comparisons of cerebral cortical measures between patients with subcortical stroke and healthy controls.

### 2.5 Cortical structural changes in patients with chronic subcortical stroke

Cross‐sectional data set was used to identify cortical structural changes in patients with chronic subcortical stroke. In the discovery sample (114 patients, and 103 healthy controls of data set 1), patients were further divided into a left‐hemispheric lesion group (*n* = 60) and a right‐hemispheric lesion group (*n* = 54), which were separately compared with the control group (*n* = 103). For each patient group, a general linear model was used to perform vertex‐based comparisons between patients and controls for cortical thickness, surface area, and GMV within a cortical mask. Age, sex, scanner variables, mean cortical thickness (only for cortical thickness analysis), total surface area (only for surface area analysis), and total GMV (only for GMV analysis) of the entire cerebral cortex were entered as nuisance covariates. Multiple comparisons were corrected using a Monte Carlo simulation with a voxel threshold of *p* < .01 and a correction threshold of *p* < .05 (1000 simulations, full‐width‐at‐half‐maximum = 10 mm). Vertices with significant intergroup differences in cortical measures were extracted as seed masks.

In the replication sample (67 patients, and 103 healthy controls, same as to the discovery sample), patients were also divided into left‐hemispheric (*n* = 35) and right‐hemispheric (*n* = 32) lesion groups, which were used for lesion‐side‐specific validation of the findings obtained from the discovery sample. Within each seed mask, we performed lesion‐side‐specific vertex‐based intergroup comparisons (*p* < .05) in the replication sample to identify clusters with reliable cortical structural changes in chronic patients with subcortical stroke. The same statistical model and covariates as in the discovery analysis were used in replication. Clusters significant in both discovery and replication samples were defined as regions of interest (ROIs). One‐way analysis of covariance was used to compare differences in cortical measures in each ROI among the PR, CR, and control groups at *p* < .05 with the same covariates. Cohen's *d* was used to describe the effect size (ES) of the intergroup differences.

### 2.6 Correlations between cortical structural changes and motor outcomes

In the left‐ or right‐hemispheric lesion group (cross‐sectional data set), we investigated the cortical structural changes by region that correlated with motor deficits. Specifically, we performed partial correlation analysis between cortical structural measures (cortical thickness, surface area, and GMV, each ROI) and the WE_FM scores while controlling for age, sex, and scanner variables. We performed correlation analyses a total of 10 times between cortical structural changes and motor outcomes. We used the Benjamini–Yekutieli (BY) false discovery rate (FDR) method (Benjamini & Yekutieli, 2001) (*p* < .05) to correct for multiple comparisons (https://warwick.ac.uk/fac/sci/statistics/staff/academic-research/nichols/software/fdr). The raw *p* values were BY‐FDR corrected in MATLAB (MATrix LABoratory). The positive dependence correction causes these BY‐FDRs to be closer to 1, or more conservative (Murray & Blume, 2021).

### 2.7 Correlations between cortical structural changes and lesion locations

In the left‐ or right‐hemispheric lesion group (cross‐sectional data set), VLSM (Bates et al., 2003) was used to assess the relation between acute stroke lesion locations and chronic cortical structural changes. DWI has good sensitivity for acute cerebral ischemia, and can accurately describe the location, morphology, and size of stroke lesions. Therefore, stroke lesions were manually delineated on the normalized DWI acquired in the acute stage. First, individual DWI data were spatially normalized to the EPI template in Montreal Neurological Institute (MNI) space and resampled into 1‐mm^3^ voxels. Stroke lesions were independently outlined on the normalized DWI using the MRIcron tool (https://www.nitrc.org/projects/mricron) by three radiologists with more than 9 years of experience. The intraclass correlation coefficient for lesion volume was 0.98, and the result of the most senior radiologist was selected as the final lesion contour.

VLSM was performed on the lesion maps against the cortical structural measures of the ROIs in the chronic stage of stroke, with age, sex, lesion volume, and scanner variables as covariates. In this analysis, we only included stroke lesion voxels that were damaged in more than 10% of the patients (Timpert et al., 2015) and only reported stroke lesion clusters that showed significant correlation in more than 10 lesion voxels (McDonald et al., 2017). Correction for multiple comparisons was achieved using a voxel‐level FDR method (*p* < .05, FDR corrected) (Raphaely‐Beer et al., 2020).

### 2.8 Correlations between cortical structural changes and early impairment of CST fibers

In the left‐ or right‐hemispheric lesion group (cross‐sectional data set), we performed correlations between early impairment of CST fibers with different cortical origins and chronic cortical structural changes to identify which CST fibers influenced chronic cortical structural changes. Imaging data on acute stroke lesions and the fine‐grained map of the CST fibers published by Liu, Wang, et al. (2020) were used to calculate the percent impairment of each CST fiber class for each patient in the acute stage. For each axial slice showing an overlap between the stroke lesion and a given CST fiber subset, an impairment percentage was calculated as the ratio of the area of the tract in the overlap region to the total area of the CST fiber subset. The largest percentage in these slices was defined as the impairment percentage of the CST fiber in this patient. We then investigated the correlations between early impairment of each CST fiber subset and cortical structural changes in the chronic stage, while controlling for age, sex, scanner variables, and the impairment percentages of other CST subsets. We performed 40 times correlation analyses between cortical structural changes and early CST impairment. To reduce the number of false‐positive findings, we used the BY‐FDR method (*p* < .05) to correct for multiple comparisons.

### 2.9 Evolution of cortical structural changes after stroke

For each ROI with significant cortical structural change in the chronic stage, we used a linear mixed‐effects model to investigate the evolution patterns of the cortical structural changes in PR patients, CR patients, and healthy controls (longitudinal data set, 81 patients and 10 healthy controls). The random intercept term accounts for the correlation due to repeated measurements within a single patient (Gibbons et al., 1988). This model allows us to make maximum use of all available data from each patient, even if some time points are missing. All patients are assumed to have a common slope (fixed effect) and only the intercepts are allowed to vary (random effect). The model parameters were estimated by the restricted maximum likelihood method and considered significant if the *p* values were less than .05. In healthy controls, we characterized the trajectories of these cortical structural changes to establish references to identify stroke‐induced changes. In each patient group, we identified significant longitudinal cortical structural changes by assessing the significance of the slopes (*p* < .05, BY‐FDR corrected). For each ROI with chronic cortical structural changes, we investigated the differences in the evolution patterns by comparing the slopes between every pair selected from the PR, CR, and control groups (*p* < .05, BY‐FDR corrected).

**FIGURE 1:**
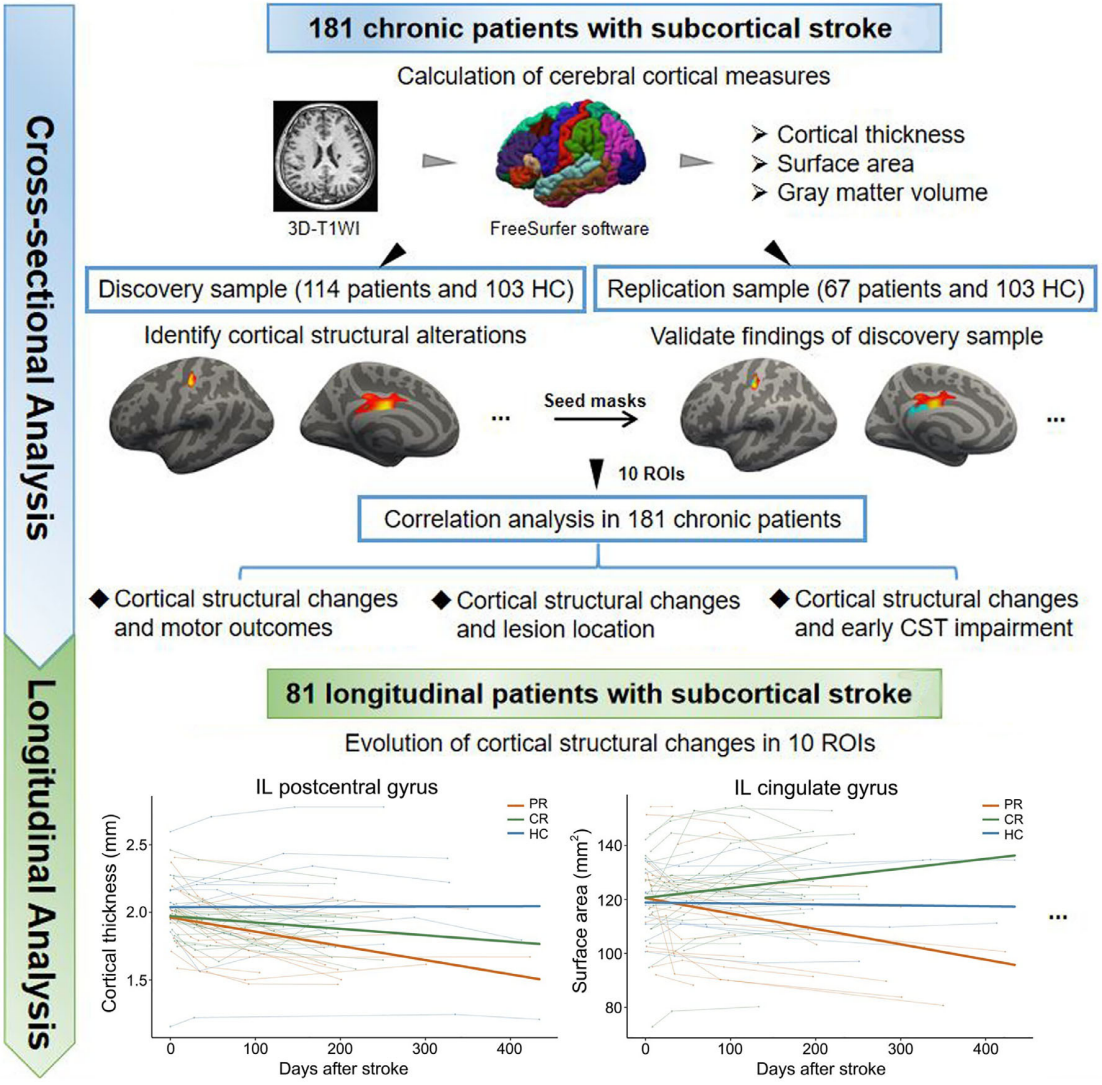
Flow diagram of the analysis. CR, complete recovery; CST, corticospinal fibers; HC, healthy control; IL, ipsilesional; PR, partial recovery; ROI, regions of interest

To show the multistep nature of the analyses, we provide a flow diagram in Figure 1.

## 3 RESULTS

### 3.1 Demographic and clinical information

**FIGURE 2:**
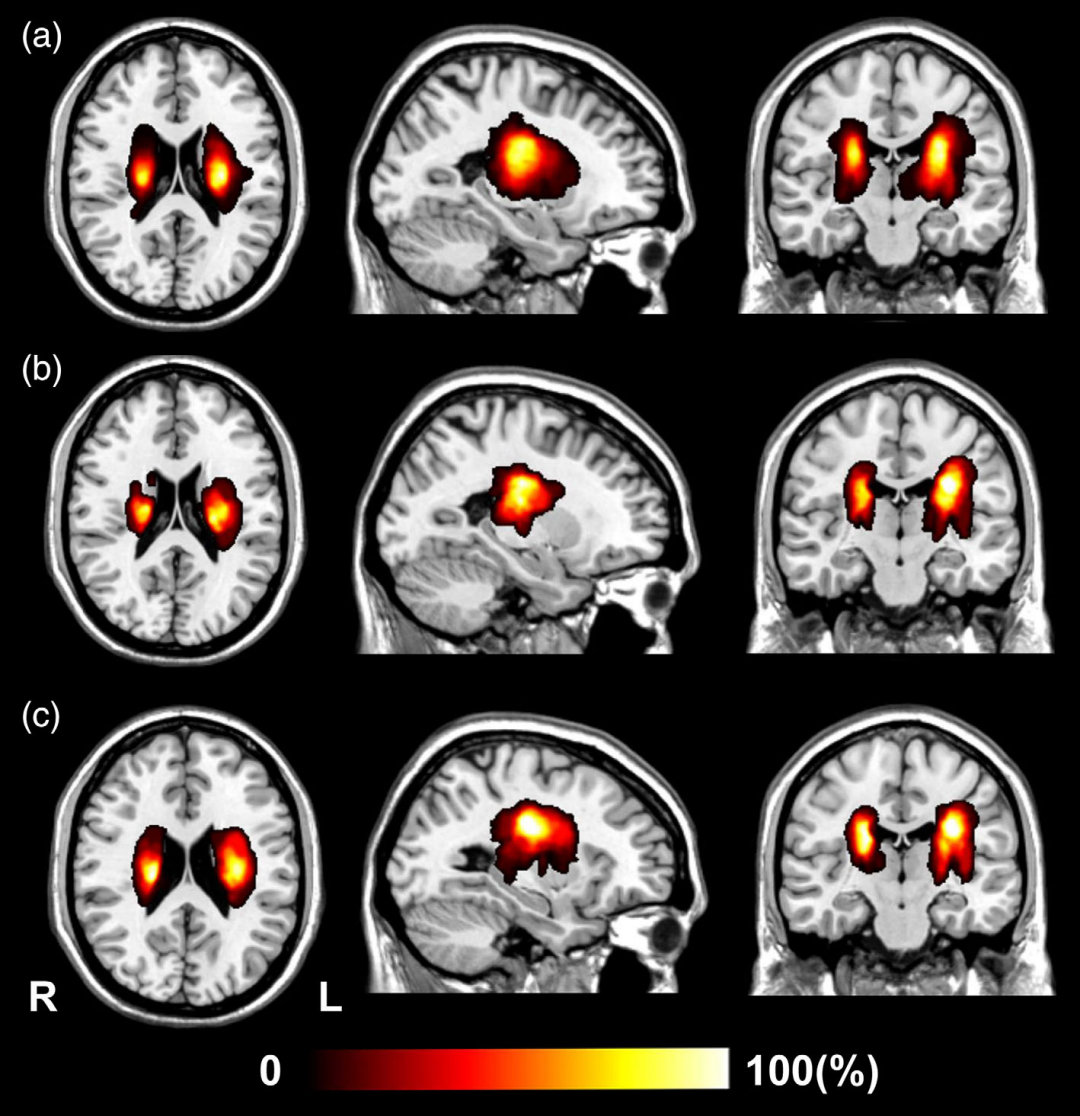
Lesion incidence map of patients with subcortical stroke. (a) For 114 patients with acute subcortical stroke (discovery sample of cross‐sectional data set). (b) For 67 patients with acute subcortical stroke (replication sample of cross‐sectional data set). (c) For 81 patients with acute subcortical stroke (longitudinal data set). L, left; R, right

The demographic and clinical data of the participants are listed in Table 1. Cross‐sectional data set included 181 patients with subcortical stroke (131 men; mean age, 55.5 ± 8.1 years; 114 patients for discovery and 67 patients for replication) and 103 healthy controls (58 men; mean age, 56.1 ± 7.3 years). We collected DWI and conventional MRI data in the acute stage as well as 3D‐T1WI and WE_FM data in the chronic stage for these patients. Longitudinal data set included 81 patients with subcortical stroke (61 men; mean age, 53.9 ± 8.9 years) and 10 healthy controls (3 men; mean age, 55.9 ± 5.2 years). We collected DWI, 3D‐T1WI, and WE_FM data from the acute to the chronic stage. The stroke lesions involved the internal capsule and the surrounding structures, including the internal capsule, thalamus, basal ganglia, and corona radiata (Figure 2a, discovery; Figure 2b, replication; Figure 2c, longitudinal data set). In cross‐sectional data set, 95 patients had stroke lesions in the left hemisphere and 86 in the right hemisphere. In longitudinal data set, 42 patients had stroke lesions in the left hemisphere and 39 in the right hemisphere.

**TABLE 1:** Demographic and clinical information of participants

Variables	Patients with subcortical stroke			Healthy controls	
	Cross‐sectional data set (*n* = 181)		Longitudinal data set (*n* = 81)	Data set 1 (*n* = 103)	Data set 2 (*n* = 10)
	Discovery (*n* = 114)	Replication^b^ (*n* = 67)		Discovery/replication	
Age, years	56.3 ± 7.8 (40–75)	54.0 ± 8.5 (30–75)	53.9 ± 8.9 (30–75)	56.1 ± 7.3 (40–75)	55.9 ± 5.2 (48–66)
Sex (M/F)	80/34	51/16	61/20	58/55	3/7
Education, years	10.3 ± 3.0 (2–19)	10.4 ± 4.1 (2–19)	9.5 ± 3.4 (2–16)	11.4 ± 2.7 (2–16)	10.0 ± 2.7 (5–12)
Time points					
1, days	2 (2–4)	2 (2–5)	5 (2–6)		0 (0–0)
2, days			35 (30.5–40)		44.5 (33.2–59)
3, months			3.3 (3.1–3.5)		4.9 (4.3–6.5)
4, months	17.1 (10.7–22)	15.6 (9.7–20)	6.6 (6.2–7.5)		8.3 (6.9–12.3)
Lesion location					
Left hemisphere	60 (52.6%)	35 (52.2%)	42 (51.9%)		
Right hemisphere	54 (47.4%)	32 (47.8%)	39 (48.1%)		
NIHSS^a^	1 (0–2)	1 (0–2)	1 (0–2)		
WE_FM^a^	91.1 ± 18.4 (19–100)	92.3 ± 16.5 (41–100)	95.9 ± 12.5 (42–100)		

*Note*: Data are presented as the means ± SD (range) for continuous data, median (Q1–Q3) for time points and NIHSS and *n* (%) for categorical data.

Abbreviations: NIHSS, National Institutes of Health Stroke Scale; WE_FM, Fugl‐Meyer Assessment of the whole extremity.

^a^ The rendered scores for clinical assessments are referred to the chronic stage of stroke.

^b^ Replication included 59 patients with chronic data from longitudinal data set.

### 3.2 Cortical structural changes in patients with chronic subcortical stroke

**FIGURE 3:**
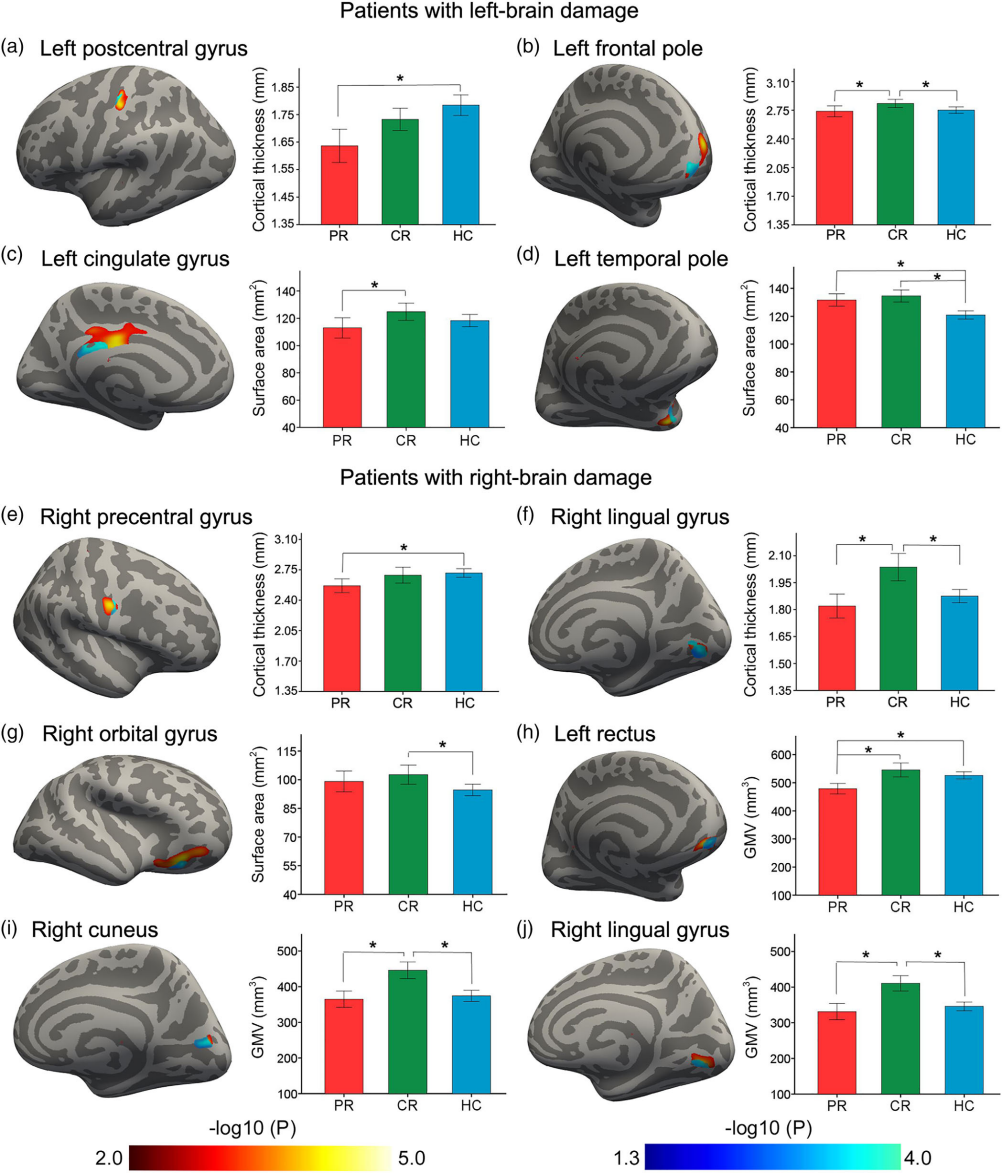
Chronic cortical structural changes in patients with subcortical stroke. In chronic patients with left subcortical stroke lesions (a–d), cortical regions with significant differences in structural measures between the patient and control groups are shown in the pial surface of the brain (left). These cortical structural measures are compared between every two of the PR, CR, and HC groups (right). In chronic patients with right subcortical stroke lesions (e–j), cortical regions with significant differences in structural measures between the patient and control groups are shown in the pial surface of the brain (left). These cortical structural measures are compared between every two of the PR, CR, and HC groups (right). Red color indicates cortical regions with intergroup difference in the discovery sample; and light blue color represents cortical regions with intergroup difference in the replication sample. CR, complete recovery; GMV, gray matter volume; HC, healthy control; PR, partial recovery

In the discovery sample, we found 10 cortical regions with significant structural differences (4 in cortical thickness, 3 in surface area, and 3 in GMV) between chronic stroke patients and healthy controls (red color in Figure 3; Table 2). All cortical structural differences were validated in the replication sample (light blue color in Figure 3; Table 2). We also compared differences in the identified ROI‐specific cortical measures among the CR, PR and control groups (bar charts in Figure 3).

**TABLE 2:** Chronic cortical structural changes in patients with subcortical stroke

Measures and regions	Discovery sample (*n* = 114)		Replication sample (*n* = 67)	
	MNI coordinates (x, y, and z)	Cluster size (mm^2^)	MNI coordinates (x, y, and z)	Cluster size (mm^2^)
*Patients with left‐brain damage*				
Cortical thickness				
Left postcentral gyrus	−45.4, −22.1, 56.8	207	−42.3, −22.3, 53.4	39
Left frontal pole	−16.7, 59.2, 3.3	390	−6.9, 61.3, −8.2	126
Surface area				
Left cingulate gyrus	−1.3, −12.8, 26.5	657	−6.1, −39.1, 22.6	151
Left temporal pole	−26.2, 5.8, −33	261	−33.2, 11.4, −29.8	103
*Patients with right‐brain damage*				
Cortical thickness				
Right precentral gyrus	48.9, −4.5, 31.5	220	52.3, −2.9, 36.3	36
Right lingual gyrus	11.8, −80.4, −4	478	12.3, −79.3, −5.4	404
Surface area				
Right orbital gyrus	30.5, 28.2, −12.3	621	20.4, 22.2, −14.6	80
Gray matter volume				
Left rectus	−5.5, 52.2, −17.7	246	−7, 54.9, −19.3	129
Right cuneus	3.9, −74.8, 16.2	242	7.1, −72.8, 16.1	203
Right lingual gyrus	11.9, −78.6, −0.9	520	14.6, −73.1, −1.7	181

Abbreviation: MNI, Montreal Neurological Institute.

In chronic patients with a left‐hemispheric lesion, PR patients showed cortical thickness reduction (*p* = .009, ES = 0.4) in the left postcentral gyrus (peak MNI coordinate = −42.3, −22.3, 53.4; cluster size = 39 mm^2^) compared with healthy controls (Figure 3a); CR patients had increased cortical thickness in the left frontal pole (peak MNI coordinate = −6.9, 61.3, −8.2; cluster size = 126 mm^2^) compared with PR patients (*p* = .028, ES = 0.4) and healthy controls (*p* = .014, ES = 0.3; Figure 3b); PR patients exhibited surface area reduction (*p* = .019, ES = 0.4) in the left cingulate gyrus (peak MNI coordinate = −6.1, −39.1, 22.6; cluster size = 151 mm^2^) compared with CR patients (Figure 3c); and both PR patients and CR patients demonstrated increased surface area compared with healthy controls (*p* = 9.9 × 10^−4^, ES = 0.5; and *p* = 3.3 × 10^−7^, ES = 0.6; respectively) in the left temporal pole (peak MNI coordinate = −33.2, 11.4, −29.8; cluster size = 103 mm^2^; Figure 3d).

In chronic stroke patients with a right‐hemispheric lesion, PR patients showed cortical thickness reduction (*p* = .009, ES = 0.4) in the right precentral gyrus (peak MNI coordinate = 52.3, −2.9, 36.3; cluster size = 36 mm^2^) compared with healthy controls (Figure 3e); CR patients exhibited increased cortical thickness in the right lingual gyrus (peak MNI coordinate = 12.3, −79.3, −5.4; cluster size = 404 mm^2^) compared with PR patients (*p* = 1.4 × 10^−5^, ES = 0.7) and healthy controls (*p* = 9.6 × 10^−5^, ES = 0.5; Figure 3f). CR patients had increased surface area (*p* = .02, ES = 0.4) in the right orbital gyrus (peak MNI coordinate = 20.4, 22.2, −14.6; cluster size = 80 mm^2^) compared with healthy controls (Figure 3g); PR patients demonstrated a GMV reduction in the left rectus (peak MNI coordinate = −7, 54.9, −19.3; cluster size = 129 mm^2^) compared with CR patients (*p* = 4.7 × 10^−5^, ES = 0.7) and healthy controls (*p* = 6.9 × 10^−4^, ES = 0.5; Figure 3h); CR patients showed increased GMV in the right cuneus (peak MNI coordinate = 7.1, −72.8, 16.1; cluster size = 203 mm^2^) compared with PR patients (*p* = 1.5 × 10^−5^, ES = 0.8) and healthy controls (*p* = 3.0 × 10^−6^, ES = 0.6; Figure 3i); and CR patients had increased GMV in the right lingual gyrus (peak MNI coordinate = 14.6, −73.1, −1.7; cluster size = 181 mm^2^) compared with PR patients (*p* = 5.9 × 10^−7^, ES = 0.8) and healthy controls (*p* = 7.9 × 10^−7^, ES = 0.7; Figure 3j).

### 3.3 Correlations between cortical structural changes and motor outcomes

Table 3 shows the correlations observed between the cortical structural measures of ROIs with significant changes in the chronic stage and motor outcomes in 181 patients with chronic subcortical stroke. In patients with a left‐hemispheric lesion, the WE_FM scores were positively correlated with the cortical thickness of the left postcentral gyrus and frontal pole and with the surface area of the left cingulate gyrus (*p* < .05). In patients with a right‐hemispheric lesion, the WE_FM scores were positively correlated with the cortical thicknesses of the right precentral and lingual gyri, and with the GMV of the left rectus, right cuneus, and lingual gyrus (*p* < .05). However, these correlations did not survive the BY‐FDR correction for multiple comparisons.

**TABLE 3:** Correlations between chronic cortical structural changes and motor outcomes in patients with subcortical stroke (cross‐sectional data set)

Regions	*pr*/uncorrected *p*	FDR corrected *p*
*Patients with left‐brain damage*		
Cortical thickness		
Left postcentral gyrus	0.230/0.028	0.1
Left frontal pole	0.286/0.0056	0.1
Surface area		
Left cingulate gyrus	0.216/0.039	0.2
Left temporal pole	0.111/0.3	1.0
*Patients with right‐brain damage*		
Cortical thickness		
Right precentral gyrus	0.268/0.014	0.1
Right lingual gyrus	0.225/0.041	0.2
Surface area		
Right orbital gyrus	0.044/0.7	1.0
Gray matter volume		
Left rectus	0.240/0.029	0.1
Right cuneus	0.245/0.025	0.1
Right lingual gyrus	0.241/0.028	0.1

*Note*: Data are presented as the partial correlation coefficient (*pr*), uncorrected and FDR corrected *p* value.

Abbreviation: FDR, false discovery rate.

### 3.4 Correlations between cortical structural changes and lesion locations

**FIGURE 4:**
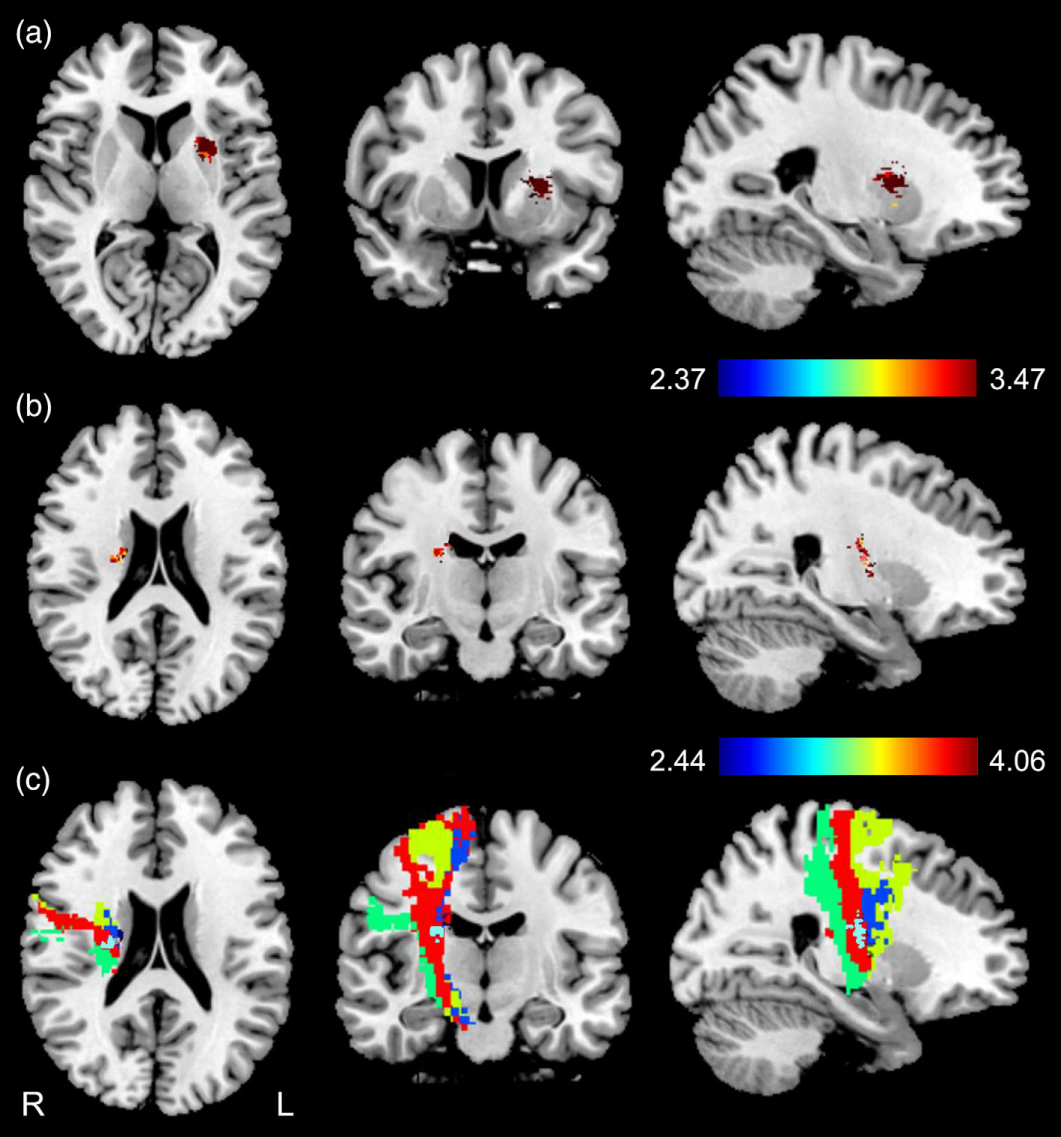
Associations between acute lesion locations and chronic cortical structural changes in patients with subcortical stroke. (a) VLSM shows that acute stroke lesion involved in the left putamen is correlated with the surface area of the left temporal pole in chronic patients with left subcortical stroke lesions. Color bar denotes the *t* values. (b) VLSM shows that acute stroke lesion involved in the right M1 fibers is correlated with the cortical thickness of the right precentral gyrus in chronic patients with right subcortical stroke lesions. Color bar denotes the *t* values. (c) Responsible lesion (light blue color) involved in the right M1 fibers, basing on a fine map of corticospinal tract (CST) fibers with different cortical origins (red for M1 fiber; yellow for PMC fiber; green for S1 fiber; and blue for SMA fiber). L, left; M1, primary motor area; PMC, premotor cortex; R, right; S1, primary sensory area; SMA, supplementary motor area; VLSM, voxel‐based lesion‐symptom mapping

In patients with lesions in the left hemisphere (cross‐sectional data set), acute‐stroke lesions in the left putamen (center MNI coordinates: −23, 5, 11; peak MNI coordinates: −19, 2, 19; cluster volume: 965 ml) were correlated with increased surface area in the left temporal pole in the chronic stage (*p* < .05, FDR correction; Figure 4a). In patients with lesions in the right hemisphere (cross‐sectional data set), acute‐stroke lesions in the right CST fibers originating from the primary motor area (center MNI coordinates: 20, −9, 21; peak MNI coordinates: 23, −12, 20; cluster volume: 422 ml) were correlated with reduced cortical thickness in the right precentral gyrus in the chronic stage (*p* < .05, FDR correction; Figure 4b,c). Other brain structural changes were not significantly correlated with lesion locations.

### 3.5 Correlations between cortical structural changes and early CST impairment

For 181 patients with chronic subcortical stroke, Table 4 shows the correlations of cortical structural changes in the chronic stage with the impairment percentages of each subset of CST fibers in the acute stage. In patients with lesions in the right hemisphere, the cortical thickness of the right lingual gyrus in the chronic stage was positively correlated with the impairment percentage of S1‐originated fibers in the acute stage (*pr* = 0.543, *p* = .024, BY‐FDR correction; Supplementary Figure 1). Although we observed additional significant correlations (*p* < .05, uncorrected), they failed to pass the BY‐FDR correction for multiple comparisons.

**TABLE 4:** Correlations between early CST impairments and chronic cortical structural changes in patients with subcortical stroke (cross‐sectional data set)

Regions	M1	PMC	SMA	S1
*Patients with left‐brain damage*				
Cortical thickness				
Left postcentral gyrus	0.3/1.0	0.1/1.0	0.1/1.0	2.3 × 10^−3^/0.1
Left frontal pole	5.2 × 10^−2^/1.0	0.6/1.0	0.7/1.0	8.9 × 10^−3^/0.4
Surface area				
Left cingulate gyrus	0.4/1.0	0.9/1.0	0.9/1.0	0.4/1.0
Left temporal pole	0.4/1.0	0.4/1.0	0.6/1.0	0.4/1.0
*Patients with right‐brain damage*				
Cortical thickness				
Right precentral gyrus	0.3/1.0	1.2 × 10^−2^/0.4	5.5 × 10^−2^/1.00.8/1.0	0.4/1.0
Right lingual gyrus	0.7/1.0	0.8/1.0	0.8/1.0	1.4 × 10^−4^/**2.4 × 10** ^−2^*
Surface area				
Right orbital gyrus	0.6/1.0	0.9/1.0	0.6/1.0	0.9/1.0
Gray matter volume				
Left rectus	6.1 × 10^−2^/1.0	8.7 × 10^−2^/1.0	2.5 × 10^−3^/0.1	0.7/1.0
Right cuneus	0.9/1.0	0.8/1.0	0.9/1.0	0.2/1.0
Right lingual gyrus	0.9/1.0	0.5/1.0	0.2/1.0	6.4 × 10^−2^/1.0

*Note*: Data are presented as the uncorrected/FDR corrected *p* value. Bold value and the star (*) means that the result can be survived after FDR correction (*p < .05).

Abbreviations: CST, corticospinal tracts; FDR, false discovery rate; M1, primary motor cortex; PMC, premotor cortex; S1, primary sensory cortex; SMA, supplementary motor area.

### 3.6 Evolution of cortical structural changes after subcortical stroke

**FIGURE 5:**
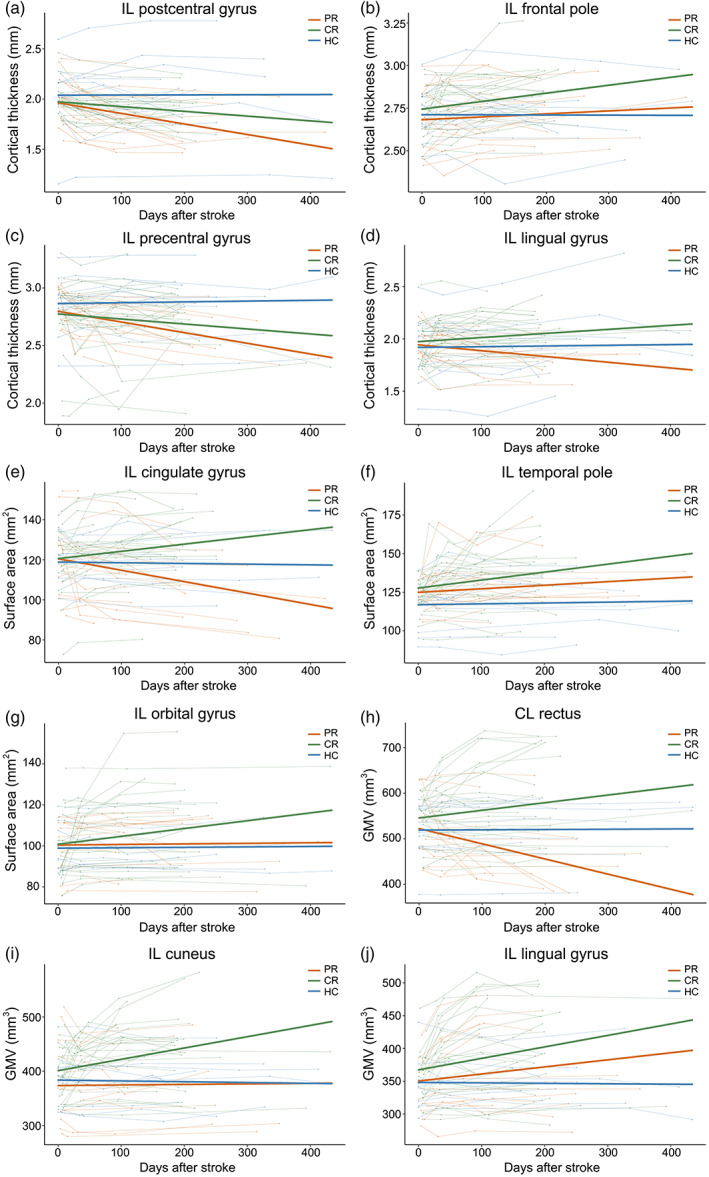
Trajectories of cortical structural changes in patients with subcortical stroke. (a–j) The longitudinal evolutionary trajectories of structural measures in cortical regions with significant chronic structural changes in patients with subcortical stroke. The thin red (PR), green (CR), and blue (HC) lines represent individual cortical structural changes over time; and the thick red, green, and blue lines indicate the estimated average cortical structural changes of the three groups. CL, contralesional; CR, complete recovery; GMV, gray matter volume; HC, healthy controls; IL, ipsilesional; PR, partial recovery

In 81 subcortical stroke patients with longitudinal brain structural MRI data (longitudinal data set), we observed longitudinal cortical structural changes in 10 ROIs that showed significant structural differences between patients with chronic subcortical stroke and healthy controls (cross‐sectional data set). The trajectories of cortical structural changes in these ROIs are shown in Figure 5. The horizontal axis represents the actual acquisition time, which was fluctuated based on four time points. The statistical significance of the longitudinal cortical structural changes at each ROI in the PR, CR, and control groups are provided in Table 5, as well as the intergroup slope differences in these changes. In the cortical thickness of the ipsilesional postcentral gyrus (Figure 5a), only the PR group showed significant longitudinal change (decline over time; *p* = 2.0 × 10^−4^, BY‐FDR correction) and showed a steeper slope (*p* = .031, BY‐FDR correction) than did the control group. In the cortical thickness of the ipsilesional frontal pole (Figure 5b), only the CR group showed significant longitudinal change (increase over time; *p* = .004, BY‐FDR correction), but the slopes did not differ between groups. In the cortical thickness of the ipsilesional precentral gyrus (Figure 5c), only the PR group showed significant longitudinal change (decline over time; *p* = .002, BY‐FDR correction) and had a steeper slope (*p* = .012, BY‐FDR correction) than did the control group. In the cortical thickness of the ipsilesional lingual gyrus (Figure 5d), we did not observe significant longitudinal changes (*p* > .05, BY‐FDR correction); however, the change direction was different between the CR group (increase) and the PR group (decrease) and their slopes were significantly different (*p* = .04, BY‐FDR correction). In the surface area of the ipsilesional cingulate gyrus (Figure 5e), the PR group (decline over time; *p* = 3.5 × 10^−5^, BY‐FDR correction) and CR group (increase over time; *p* = .005, BY‐FDR correction) showed diverging trajectories, and the slope of the PR group was significantly different from that of the CR group (*p* = 2.0 × 10^−7^, BY‐FDR correction) and control group (*p* = .018, BY‐FDR correction). In the surface area of the ipsilesional temporal pole (Figure 5f), only the CR group showed significant longitudinal change (increase over time; *p* = .031, BY‐FDR correction), but the slopes did not differ between groups. In the surface area of the ipsilesional orbital gyrus (Figure 5g), no significant longitudinal changes or slope differences were observed (*p* > .05, BY‐FDR correction). In the GMV of the contralesional rectus (Figure 5h), only the PR group showed significant longitudinal change (decline over time; *p* = .005, BY‐FDR correction) and its slope was significantly different from that of the CR group (*p* = 2.0 × 10^−4^, BY‐FDR correction). In the GMV of the ipsilesional cuneus (Figure 5i), only the CR group showed significant longitudinal change (increase over time; *p* = .004, BY‐FDR correction), but the slopes did not differ between groups. In the GMV of the ipsilesional lingual gyrus (Figure 5j), only the CR group showed significant longitudinal change (increase over time; *p* = 3.5 × 10^−5^, BY‐FDR correction), and its slope was significantly greater than that of the control group (*p* = .033, BY‐FDR correction).

**TABLE 5:** Longitudinal evolution patterns of cortical structural changes after subcortical stroke (longitudinal data set)

Regions	PR	CR	HC	PR vs. CR	CR vs. HC	PR vs. HC
*Cortical thickness*						
IL postcentral gyrus	3.6 × 10^−6^/**2.0 × 10** ^−4^*	9.5 × 10^−3^/0.1	0.9/1.0	4.4 × 10^−2^/0.4	0.1/0.9	1.6 × 10^−3^/**3.1 × 10** ^−2^*
IL frontal pole	0.2/1.0	1.3 × 10^−4^/**4.4 × 10** ^−3^*	0.9/1.0	0.1/0.9	1.3 × 10^−2^/0.2	0.4/1.0
IL precentral gyrus	3.1 × 10^−5^/**1.5 × 10** ^−3^*	4.5 × 10^−3^/7.0 × 10^−2^	0.7/1.0	6.4 × 10^−2^/0.6	3.1 × 10^−2^/0.3	4.7 × 10^−4^/**1.2 × 10** ^−2^*
IL lingual gyrus	2.9 × 10^−2^/0.3	2.4 × 10^−2^/0.3	0.8/1.0	2.3 × 10^−3^/**4.0 × 10** ^−2^*	0.3/1.0	7.5 × 10^−2^/0.7
*Surface area*						
IL cingulate gyrus	2.9 × 10^−7^/**3.5 × 10** ^−5^*	1.8 × 10^−4^/**5.1 × 10** ^−3^*	0.8/1.0	6.7 × 10^−10^/**2.0 × 10** ^−7^*	8.1 × 10^−3^/0.1	7.6 × 10^−4^/**1.8 × 10** ^−2^*
IL temporal pole	0.2/1.0	1.5 × 10^−3^/**3.1 × 10** ^−2^*	0.8/1.0	0.3/1.0	8.3 × 10^−2^/0.7	0.5/1.0
IL orbital gyrus	0.9/1.0	4.7 × 10^−3^/6.9 × 10^−2^	0.9/1.0	0.1/1.0	0.1/1.0	0.9/1.0
*Gray matter volume*						
CL rectus	1.5 × 10^−4^/**4.6 × 10** ^−3^*	4.5 × 10^−3^/6.9 × 10^−2^	0.9/1.0	3.1 × 10^−6^/**2.0 × 10** ^−4^*	0.1/1.0	6.4 × 10^−3^/0.1
IL cuneus	0.9/1.0	1.2 × 10^−4^/**4.4 × 10** ^−3^*	0.9/1.0	3.8 × 10^−2^/0.4	2.0 × 10^−2^/0.2	0.8/1.0
IL lingual gyrus	2.6 × 10^−2^/0.3	3.8 × 10^−7^/**3.5 × 10** ^−5^*	0.9/1.0	0.3/1.0	1.8 × 10^−3^/**3.3 × 10** ^−2^*	8.9 × 10^−2^/0.7

*Note*: Data are presented as the uncorrected/FDR corrected *p* value. Bold values and the star (*) means that the result can be survived after FDR correction (*p < .05).

Abbreviations: CL, contralesional; CR, complete recovery; FDR, false discovery rate; HC, healthy controls; IL, ipsilesional; PR, partial recovery; vs, versus.

## 4 DISCUSSION

In this study, we investigated chronic cortical structural changes and their evolution patterns in subcortical stroke patients with different degrees of motor recovery, elucidating the correspondence of these changes with the locations of lesions and the early impairment of specific CST fiber subsets. We found that chronic cortical structural changes and their evolution patterns were largely different between partially and completely recovered patients. We linked some chronic cortical structural changes to specific lesion locations and to the early impairment of specific CST fiber subsets. These findings indicate that early brain impairments suggested by lesion locations are predictive of chronic cortical structural changes, and that the evolution patterns of certain cortical structural changes are predictive of long‐term motor recovery in these patients.

### 4.1 Cortical structural measures can provide complementary information

In contrast to most previous studies, which characterized cortical structural changes using either cortical thickness or GMV (Jiang et al., 2017; Ueda et al., 2019), we used cortical thickness, surface area, and GMV to characterized cortical structural changes after subcortical stroke and found that different cortical structural measures were changed in different cortical regions. Our findings suggest that these structural measures can provide complementary information about cortical structural changes (Fornito et al., 2008) and should be used in combination to characterize such changes (Buechler et al., 2020).

GMV is a composite indicator, being affected by both cortical thickness and surface area. For example, a simultaneous significant cortical thickness reduction and surface area increase may result in a nonsignificant GMV change; however, a slightly reduced cortical thickness with a slightly reduced surface area may lead to a significant GMV reduction. Cortical thickness measures distances between the gray/white matter interface and the pial surface (the surface between gray matter and cerebrospinal fluid). Cortical area measures the area of the gray‐matter surface. Cortical thickness and surface area are two independent dimensions that reflect the microstructural characteristics of the cortex, and multidimensional measurements are thus preferred for noninvasively capturing microstructural changes after stroke. However, the developmental and reorganizational trajectories of cortical thickness and surface area differ (Wierenga et al., 2014), differing in both onset and timing and by anatomical region (Brodtmann et al., 2012). This situation may contribute to the result that one measure may show differences after stroke while another may not.

### 4.2 Subcortical stroke can result in both cortical structural damage and reorganization

Consistent with previous studies (Jones et al., 2016; Liu, Peng, et al., 2020; Zhang et al., 2014), chronic patients with subcortical stroke showed both cortical structural damage and reorganization. In these patients, the most consistent cortical structural damage is damage to the sensorimotor cortex in the hemisphere ipsilateral to the lesion (Jones et al., 2016; Zhang et al., 2014). In this study, we also found cortical thinning in the right precentral gyrus in patients with right subcortical lesions and in the left postcentral gyrus in patients with left subcortical lesions. The structural damage in the ipsilesional sensorimotor cortex may be explained by antegrade and/or retrograde axonal degeneration (Yu et al., 2009), since the subcortical stroke lesions can directly impair the output or input fibers of the sensorimotor cortex or impair brain regions that are connected with the sensorimotor cortex (Duering et al., 2012; Duering et al., 2015). However, the structural damage observed in the left rectus in patients with right subcortical lesions cannot be easily explained by the mechanism of axonal degeneration and requires further clarification.

The structural reorganization of the cerebral cortex was scattered through frontal, occipital, and temporal lobes remote from the stroke lesions. The structural reorganization in the prefrontal cortex found in previous studies (Fan et al., 2013; Jiang et al., 2017; Liu, Peng, et al., 2020) and in this study (frontal pole and orbitofrontal gyrus) indicate that cognitive‐related cortical regions may be involved in motor recovery and that cognitive strategy may play a beneficial role in motor recovery in patients with subcortical stroke (McEwen et al., 2009). In line with prior studies (Al Harrach et al., 2019; Fan et al., 2013), we also found structural reorganization in several occipital cortical regions. Although the functional significance and neural mechanism of occipital cortical reorganization after subcortical stroke remain unknown, occipital reorganization may be related to motor recovery since these cortical changes were marginally correlated here with motor outcomes (*p* < .05, uncorrected).

### 4.3 Cortical structural changes depend on lesion location in subcortical stroke

In this study, stroke patients with lesions in the left and right hemispheres showed structural changes in different cortical regions, which is consistent with the great variation in chronic cortical structural changes following subcortical stroke reported across studies (Diao et al., 2017; Jiang et al., 2017; Liu, Peng, et al., 2020). Lesion locations largely differ among patients with subcortical stroke, which may be an important cause of the variation in the reported chronic cortical structural changes following subcortical stroke. Theoretically, stroke lesions in different anatomical locations impair different brain structures, resulting in structural changes in different cortical regions. To determine the causal relations between lesion locations and chronic cortical structural changes, we investigated the correlations of the latter with acute stroke lesion locations and early impairment of the CST fibers. VLSM showed that right lesions involving CST fibers originating from the primary motor area resulted in cortical thinning in the right precentral gyrus, indicating that retrograde degeneration is the neural mechanism of structural damage in the ipsilesional precentral gyrus (Duering et al., 2015; Yu et al., 2009). VLSM also demonstrated that stroke lesions in the left putamen were associated with increased surface area in the left temporal pole. Although the mechanisms underlying this relation are unclear, this result may indicate that damage in a specific subcortical region can lead to structural reorganization in a specific cortical region. We used a fine‐grained map of the cortical origins of CST fibers to determine the degrees of impairment in CST fiber subsets, and uncovered the impacts of early impairments in specific CST subsets on cortical structural changes. Using the CST damage‐cortical change association study, we found that early impairment of the CST fibers originating from the primary somatosensory cortex was associated with structural reorganization in the occipital cortex. This may represent a large‐scale compensatory mechanism in which the visual system reorganizes itself to compensate for impairments in the somatosensory system (Pundik et al., 2018).

### 4.4 Patterns of cortical structural evolution are predictive of neurological recovery

Consistent with previous studies that reported correlations between cortical structural changes and motor outcomes in patients with subcortical stroke (Zhang et al., 2014), we found that the chronic cortical structural changes and their evolution trajectories were largely different between partially and completely recovered patients. Compared with healthy controls, patients with PR showed more significant cortical structural damage, but patients with CR demonstrated more significant cortical reorganization. These findings indicate that in patients with subcortical stroke, cortical structural damage and reorganization are correlated with motor outcome. In the 10 cortical regions with chronic structural changes, we observed four types of evolution pattern: pattern 1, decrease with different slopes in CR and PR patients; pattern 2, increase with different slopes; pattern 3, increase only in CR patients; and pattern 4, divergent trajectories in CR and PR patients. Pattern 1 was observed in the primary sensorimotor cortex, which is commonly damaged in stroke and is useful in predicting motor deficits. Pattern 2 was observed in the lingual gyrus, representing a common cortical structural reorganization. Pattern 3 was observed in the frontal pole, temporal pole, orbital gyrus, and cuneus. This pattern represents structural reorganization in CR patients and is an indicator of good recovery. Pattern 4 was observed in the gyrus rectus, cingulate gyrus, and lingual gyrus, and represents cortical structural reorganization in CR patients and cortical structural damage in PR patients. Pattern 4 can be used to differentiate patients with PR from those with CR.

The present study has several limitations. First, the lesion locations in our samples were restricted: just the basal ganglia and neighboring regions, it will extend to the general stroke population in the future study. Second, the same 103 healthy controls (data set 1) were used for both the discovery and replication analysis, which might affect the interpretation and generalizability of our findings, and future studies should try to use different healthy control data sets to validate this study. Third, in the VLSM analysis, individual DWI data were spatially normalized to the EPI template in MNI space, which may be potentially affected by the small lesion volumes. However, we examined the accuracy of lesion coregistration and did not find visible changes in lesion location, morphology, or size. Fourth, there are 29 patients with missing time points in longitudinal data set. Although linear mixed‐effects model allows us to use data from some missing time points, missing data points might affect our findings, and future studies should try to recruit all time points to validate this study. For stroke patients at the chronic stage, the scanning time varied greatly because of difficulties with long‐term follow‐up. Some patients were scanned at 6 months after stroke, but other patients were scanned later (>1 year). Despite the large time interval, these patients were in a stable chronic stage with little changes in motor function. In our study, these data points (≥200 days following stroke) were brought into the linear mixed‐effects model. Because the small number of data points after 200 days might have impacted our slope determinations, future studies should recruit time points at shorter intervals.

## 5 CONCLUSION

Both chronic cortical structural changes and their evolution patterns were studied in patients with subcortical stroke. The cortical changes and their evolution differed between partially and completely recovered stroke patients. This effect may be used to screen imaging biomarkers for predictors of motor outcomes. We established the correspondence of some cortical structural changes with the location of the acute stroke lesion and with early impairment of CST fibers with specific cortical origins. These findings are useful for developing approaches for the early prediction of motor outcomes, which is helpful in the design of individualized rehabilitation plans.

## AUTHOR CONTRIBUTIONS

Jingchun Liu and Caihong Wang contributed to conception and design of the study. Wen Qin, Hao Ding, and Yanmin Peng performed the experiments and analyzed the data. Jun Guo and Tong Han were involved in the clinical assessment. Chunshui Yu and Jingliang Cheng provided guidance and advice. Jingchun Liu, Caihong Wang, and Chunshui Yu drafted and revised the paper.

## FUNDING INFORMATION

This study was supported by the National Key Research and Development Program of China (2018YFC1314300), the Natural Science Foundation of China (81871327 and 82030053), the Tianjin Key Technology R&D Program (17ZXMFSY00090), and the Young Talents Promotion Program of Henan Province (2021HYTP012).

## CONFLICT OF INTEREST

The authors declare no competing conflict of interests.

## Supporting information


**APPENDIX S1** Supporting information

## Data Availability

The data that support the findings of this study are available on request from the corresponding author. The data are not publicly available as they contain information that could compromise the privacy of the research participants.
