# Supplementary material for: Cortical structural changes after subcortical stroke: Patterns and correlates
Source: Hum Brain Mapp. 2022 Oct 3;44(2):727–43. doi: 10.1002/hbm.26095 (PMC9842916; doi:10.1002/hbm.26095)
Supplement: Supplementary file 1 — APPENDIX S1 Supporting information [file HBM-44-727-s001.docx]

**SUPPLEMENTAL MATERIAL**

**Supplementary Table 1. Longitudinal missing pattern of time points in patients with subcortical stroke and healthy controls.**

| Variables | TP1 | TP2 | TP3 | | TP4 |
| --- | --- | --- | --- | --- | --- |
| **Partial recovery subgroup (n = 30)** | | | | | |
| Scanning numbers | 28 (93.3%) | 23 (76.7%) | | 23 (76.7%) | 21 (70%) |
| Missing numbers | 2 (6.7%) | 7 (23.3%) | | 7 (23.3%) | 9 (30%) |
| **Complete recovery subgroup (n = 51)** | | | | | |
| Scanning numbers | 49 (96.1%) | 48 (94.1%) | | 46 (90.2%) | 44 (86.3%) |
| Missing numbers | 2 (3.9%) | 3 (5.9%) | | 5 (9.8%) | 7 (13.7%) |
| **Healthy controls (n = 10)** | | | | | |
| Scanning numbers | 10 (100%) | 10 (100%) | | 10 (100%) | 10 (100%) |
| Missing numbers | 0 (0%) | 0 (0%) | | 0 (0%) | 0 (0%) |

Data are presented as the n (%) for categorical data. TP, time point.


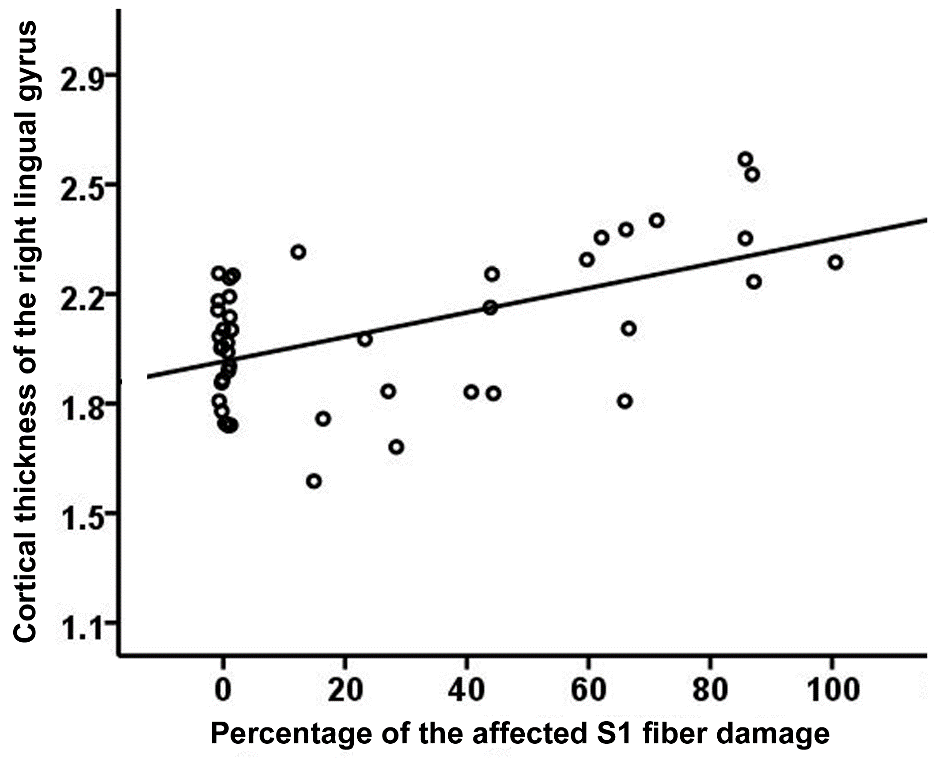


**Supplementary Figure 1. Correlations between the percentage of damage of the right corticospinal fibers originated from the primary sensory area (S1) and the cortical thickness of the right lingual gyrus in patients with subcortical stroke lesions in the right hemisphere.**
